# A single dose of exenatide had no effect on blood flow velocity in the middle cerebral artery in elderly healthy volunteers: Randomized, placebo-controlled, double-blind clinical trial

**DOI:** 10.3389/fnagi.2022.899389

**Published:** 2022-07-25

**Authors:** Joakim Ölmestig, Ida R. Marlet, Tina Vilsbøll, Jørgen Rungby, Egill Rostrup, Kate L. Lambertsen, Christina Kruuse

**Affiliations:** ^1^Neurovascular Research Unit, Department of Neurology, Copenhagen University Hospital – Herlev and Gentofte, Copenhagen, Denmark; ^2^Department of Clinical Medicine, University of Copenhagen, Copenhagen, Denmark; ^3^Steno Diabetes Center Copenhagen, Copenhagen, Denmark; ^4^Department of Endocrinology, Copenhagen University Hospital – Bispebjerg and Frederiksberg, Copenhagen, Denmark; ^5^Center for Neuropsychiatric Schizophrenia Research, Copenhagen University Hospital – Mental Health Center Glostrup, Copenhagen, Denmark; ^6^Department of Neurobiology Research, Institute of Molecular Medicine, University of Southern Denmark, Odense, Denmark; ^7^Department of Neurology, Odense University Hospital, Odense, Denmark; ^8^BRIDGE – Brain Research-Inter-Disciplinary Guided Excellence, Department of Clinical Research, University of Southern Denmark, Odense, Denmark

**Keywords:** cerebral blood flow, healthy volunteers, glucagon-like peptide 1, exenatide, clinical trial

## Abstract

**Background and aims:**

Glucagon-like peptide 1 (GLP-1) receptor agonists (GLP-1RA) are widely used for the treatment of type 2 diabetes, and recent studies indicate that they may be cardio- and neuroprotective. The safety and effect of a single dose of exenatide, a short-acting GLP-1RA, on cerebral and peripheral arterial function remain unknown.

**Methods:**

In this randomized, double-blind pilot trial, we assigned elderly healthy volunteers without diabetes and no previous history of stroke to receive a single dose of subcutaneous exenatide (5 μg) or placebo. Primary outcome was immediate changes over time in blood flow velocity of the middle cerebral arteries (V_MCA_) assessed by repeated transcranial Doppler measurements. Secondary outcomes were changes in peripheral arterial function with finger plethysmography, ankle-brachial index (ABI), and inflammatory- and endothelial-specific biomarkers.

**Results:**

Healthy volunteers (13 women and 17 men) were included: (mean ± standard deviation) age: 62 ± 8 years; body weight: 79.6 ± 12.7 kg; V_MCA_: 65.3 ± 10.7 cm/s; fasting plasma glucose: 5.5 ± 0.5 mmol/L; HbA1c: 33.9 ± 4.1 mmol/mol (5.3 ± 0.38%). No differences between exenatide and placebo group were seen regarding V_MCA_ (*p* = 0.058), systolic ABI (*p* = 0.71), plethysmography (*p* = 0.45), tumor necrosis factor (*p* = 0.33), interleukin-6 (*p* = 0.11), interleukin-1β (*p* = 0.34), vascular cell adhesion molecule 1 (*p* = 0.73), intercellular adhesion molecule 1 (*p* = 0.74), or E-selectin (*p* = 0.31). No severe adverse events were observed.

**Conclusion:**

A single dose of exenatide did not change cerebral blood flow velocity or peripheral vessel function in elderly healthy volunteers. The medication was safe to use in persons without diabetes allowing us to investigate this drug further in search of the neuroprotective mechanisms.

**Clinical Trial Registration:**

https://clinicaltrials.gov, Identifier NCT02838589.

## Introduction

In ischemic stroke, removal of the blood clot by thrombolysis or by thrombectomy are currently used as treatment in the hyper acute phase of stroke (Phipps and Cronin, 2020). Due to a narrow therapeutic time window of 4.5–24 h and various contraindications to treatment, not all patients are eligible for this treatment (Phipps and Cronin, 2020). As a result, new therapeutic options for ischemic stroke are investigated with focus on both acute, preventive, and neuroprotective effects. Evidence increasingly suggests that glucagon-like peptide 1 (GLP-1) receptor agonists (GLP-1RAs), widely used for the treatment of type 2 diabetes, may also be used as cardio- or neuroprotectants (Salcedo et al., 2012; Gault and Hölscher, 2018).

GLP-1 is an incretin hormone that increases glucose-dependent pancreatic secretion of insulin (Nauck and Meier, 2016). Multiple drugs have been developed targeting the GLP-1 receptor, and are proven effective in treating diabetes and obesity (Vilsbøll et al., 2012). Recent large clinical studies show that GLP-1RAs reduced both cardio- and cerebrovascular events in patients with diabetes, but similar effects in individuals without diabetes, are yet to be identified in studies (Sheahan et al., 2020). The pharmacological effects of GLP-1RAs are diverse and not restricted to glycemic control (Maskery et al., 2021). Other effects include reduced gastric emptying, reduced food intake *via* direct neurochemical actions in the brain, and a modulating effect on systemic inflammation (Drucker, 2018). In animal models of ischemic stroke, GLP-1RAs reduce inflammation, endothelial leakage, oxidative stress, and apoptosis, all factors involved in stroke pathophysiology (Marlet et al., 2018). GLP-1RAs may also affect blood vessels directly, although presented results vary (Marlet et al., 2018). In a systematic review, Marlet et al. (2018) showed that one of four studies on animal stroke models found cerebral blood flow (CBF) to increase after GLP-RA treatment, while the three other studies showed no CBF effects. GLP-1RA was in one study found to induce vasodilation in rat mesenteric arteries (Bangshaab et al., 2019) and to induce coronary artery dilation in humans (Clarke et al., 2018). Furthermore, it improves recruitment of microvasculature in human muscles (Sjøberg et al., 2014), apparently nitric oxide-dependent (Chai et al., 2012).

It is yet to be demonstrated by which mechanisms GLP-1RAs protect against cardio- and cerebrovascular events in diabetics or if GLP-1RAs affect the human cerebral vasculature directly. In this study, we aim to examine if a single dose GLP-1RA affects the human cerebral vasculature and changes CBF velocity in healthy elderly individuals without diabetes or cerebrovascular disease, in search of the mechanism behind the neuroprotective effect of GLP-1RAs. Further, we wanted to examine the effect of GLP-1RAs on peripheral arterial function using finger plethysmography and ankle-brachial index (ABI), and by measuring vascular and inflammatory biomarkers. We hypothesize that a single dose of GLP-1RA will change CBF velocity in the middle cerebral arteries (MCA) due to age-related endothelial dysfunction. Further, we hypothesize it will improve peripheral vascular reactivity in the fingers, improve ABI, and reduce levels of inflammatory biomarkers.

This study may improve our understanding of how GLP-1RAs affect the human cerebral and peripheral vasculature in older subjects without diabetes prior to designing larger multi center studies in stroke patients of similar age. Before the intervention and outcomes are tested in stroke patients, it is important to test it in healthy persons as a safety measure.

## Materials and methods

The trial followed guidelines for good clinical practice (GCP) and was monitored by the GCP-unit in the Capital Region, Denmark. The Ethics Committee in the Capital Region of Denmark (H-16022532), the Danish Medicines Agency (EudraCT-number: 2016-001221-14), and the Danish Data Protection Agency (j.nr.: HGH-2016-058) approved this study. The trial was registered at ClinicalTrials.gov (NCT02838589). We obtained informed consent according to the Declaration of Helsinki of 1964, as revised in 2008.

### Subjects

We included healthy volunteers above the age of 50 years with no prior history of cerebrovascular disease, diabetes, chronic heart failure (NYHA ≥ II), chronic kidney disease, or pancreatitis. Fulfillments of in- and exclusion criteria (Supplementary Material) were established by blood samples, electrocardiogram (ECG), and by a questionnaire. Volunteers were recruited between 2016 and 2017 through advertisement at the hospital and public webpages.^1^

### Design and experimental protocol

This study was conducted as a randomized, double-blind, placebo controlled, parallel-arm pilot trial. Trial eligibility was determined using in- and exclusion criteria on a screening day, where we also performed a neurological examination, obtained blood pressure and heart rate measurements (Microlife BP A100, Taipei, Taiwan), electrocardiogram (GE Healthcare MAC 5500 HD, Chicago, IL, United States), as well as a basic blood screening with same-day standard analysis. We performed a Montreal Cognitive Assessment (MoCA), National Institute of Health Stroke Scale (NIHSS), and modified Rankin Scale (mRS) screening.

**FIGURE 1:**
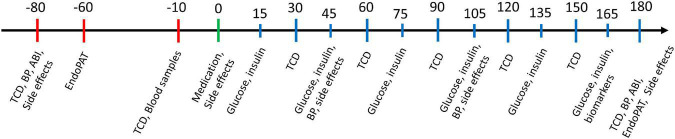
Trial day flowchart. Time shown in minutes with medication time point set to 0 min. Red bars indicate pre-medication methods, green bar indicates medication time point and blue bars indicate post-medication methods. ABI, ankle brachial index; BP, blood pressure; TCD, transcranial doppler.

On the trial day (Figure 1), patients were asked to refrain from food and drinks, except for essential medication, for 8 h prior to start of trial day (usually from midnight). Patients received a single subcutaneous dose of either 5 μg (0.05 mL) exenatide (Byetta^®^ Pen Injector, AstraZeneca) or placebo (0.9% saline water, 0.05 mL) by a trained study nurse not otherwise involved in the study. For blinding, the investigator left the examination room during administration and the participants were blindfolded during the procedure. In order to detect an immediate vascular effect, we aimed to find a compound with a quick time to maximal concentration (T_max_) and a short half-life (T_1/2_). Exenatide was chosen because of the relatively short T_1/2_ of 2.4 h. Exenatide reaches maximal plasma concentration (C_max_) after 2 h, hence we collected the final results after 3 h. Medication/placebo were randomized, packed in similar boxes, and delivered from the Capital Region Hospital Pharmacy, Denmark. The primary endpoint was changes in mean CBF velocity over time in the middle cerebral arteries (V_MCA_), measured repeatedly by transcranial Doppler (TCD). Secondary endpoints were changes in peripheral endothelial response measured by EndoPAT2000, ABI, blood pressure, serum insulin and plasma glucose, and endothelial and inflammatory biomarkers in peripheral blood samples collected from a peripheral cubital venous catheter. Biomarkers measured were E-selectin, tumor necrosis factor (TNF), interleukin (IL)-6, IL-1β, vascular cell adhesion molecule 1 (VCAM1), and intercellular adhesion molecule 1 (ICAM1). Side effects were recorded on the trial days in a dedicated questionnaire up to 24 h after drug administration corresponding to ten times T_1/2_. Trial days were performed by author IM and assisted by JÖ. The experiment was conducted with the participant in supine position on a comfortable bed in a quiet and dimly lit room at 21–24°C. A trial day lasted approximately 5 h.

### Transcranial doppler

A time-averaged mean of V_MCA_ was recorded bilaterally by TCD (2 MHz, Multidop X Doppler, DWL; Lübech and Sipplingen, Sipplingen, Germany) using handheld probes according to previously published methods (Ölmestig et al., 2020). The average of four measurements was calculated over a 30 s time interval. A fixed point for measurements of V_MCA_ was chosen along MCA as the point adjacent to the bifurcation between the MCA and the anterior cerebral artery. This fixed point was then used throughout the study in each subject, where every measurement was done after careful optimization of the signal from this point. We conducted two baseline measurements, one at 40 min pre-medication and one just before administration of medication. Measurements were then conducted every 30 min up to 3 h post-medication.

### Finger plethysmography

Endothelial function and arterial stiffness were assessed non-invasively by finger plethysmography (EndoPAT2000; Itamar Medical Ltd., Caesarea, Israel) in accordance with our previously published methods (Hansen et al., 2017; Butt et al., 2018; Ölmestig et al., 2020). Measurements were done after 30 min rest, and included a 6-min baseline, 5-min occlusion, and 4-min reactive hyperemia phase. Endothelial function was calculated by measures of pulse wave amplitude on the index finger *via* pneumatic finger probes during the reactive hyperemia phase of the forearm’s vascular bed (Kuvin et al., 2003). A reactive hyperemia index (RHI) was automatically calculated by the EndoPAT software using an automated algorithm. The values were normalized to the simultaneous signal from the control arm to reduce potential systemic effects. Arterial stiffness, calculated as an augmentation index (AI), was calculated from the arterial waveform and pulse wave amplitude during the baseline period. The augmentation index was defined as the difference between the second and first systolic peaks in the arterial waveform, expressed as a percentage of the central pulse pressure. Due to the influence of heart rate on augmentation index, the values were automatically normalized to a heart rate of 75 beats per minute (AI@75) by the EndoPAT2000 software. High AI values are an indication of stiff arteries. We performed EndoPAT recordings at baseline and 3 h post-medication.

### Blood sample analysis

We placed a peripheral cubital venous catheter (BD Venflon Pro, NJ, United States) in the dominant arm and left the arm to rest for 30 min. Baseline blood samples, including insulin and plasma glucose, were drawn just prior to administering medication/placebo. Insulin and plasma glucose samples were then drawn every 30 min post-medication. Blood for biomarker analysis was sampled a second time at the end of the trial day. Blood samples were immediately centrifuged and frozen (-40°C). Biomarkers and insulin were analyzed when all samples for all participants were collected. Plasma glucose was measured using a glucometer. We used multiplex electrochemiluminescence assay kits (Mesoscale, MD, United States) for analyzing the following biomarkers: E-selectin, TNF, IL-6, IL-1β, VCAM1, and ICAM1 (Butt et al., 2018; Ölmestig et al., 2020). Samples were diluted twofold in Diluent 41 prior to measurements and analysis. Samples were analyzed using the MSD Discovery Workbench software. The concentration for the lower limit of detection was calculated based on a signal 2.5 SD above the blank (zero) calibration value. We accepted a coefficient of variation (CV) value cut-off of 25% (Butt et al., 2018; Ölmestig et al., 2020).

### Blood pressure, heart rate, and ankle-brachial index

Blood pressure and heart rate (Microlife BP A100, Taipei, Taiwan) were measured twice at baseline and every hour after medication administration during the trial day until 3 h post-medication. ABI was chosen as measure of peripheral arterial function. Ankle blood pressure was measured with a D900 Doppler device with an 8 MHz probe (Huntleigh, Cardiff, Wales, United Kingdom) and brachial blood pressure was measured using the automatic blood pressure monitor. A value less than 0.9 were considered clinically significant.

### Statistical Analyses

All values in text are presented as mean ± standard deviation (SD) unless otherwise stated. Sample size was based on previous studies where TCD was used as primary endpoint in patients with migraine and healthy individuals (Kruuse et al., 2002, 2003, 2005; Birk et al., 2004). For the primary endpoint, a mean was calculated for each measurement at the different time points. This mean was used for further statistical analyses. Correction of data was attained by subtraction of the mean of pre-medication baseline values. A repeated-measures ANOVA for TCD, blood pressure, serum insulin, and plasma glucose were performed. An unpaired-sample *t*-test was used to analyze plethysmography, ABI, and blood biomarkers. To compare the two treatment arms, delta values for the corresponding timepoints for active and placebo treatment were calculated. Raw baseline-subtracted data and delta values were analyzed using repeated-measures ANOVA. When a significant effect of time was seen, we performed a *post hoc* analysis using unpaired-sample *t*-tests. Statistics was performed using STATA v. 13.1. A two-sided *P*-value of < 0.05 was considered statistically significant.

## Results

Thirty individuals were included and completed this trial. Subject characteristics are shown in Table 1 and results for all outcomes are presented in Tables 2, 3.

**TABLE 1:** Subject characteristics (*n* = 30).

	Exenatide mean ± SD	Placebo mean ± SD
Age (years)	61.3 ± 6.2	63.3 ± 9.1
Subjects (*n*)	15	15
Female sex (*n*)	8	5
BMI (kg/m^2^)	26.7 ± 3.8	26.1 ± 4.5
Hypertension (*n*)	4	2
Smoking status		
● Current	0	0
● Previous	8	7
● Never	7	8
Cholesterol (mmol/L)		
● Total	5.4 ± 0.9	5.3 ± 1.1
● LDL	3.0 ± 0.6	3.1 ± 0.9
● HDL	1.7 ± 0.5	1.6 ± 0.4
HbA1c (mmol/mol)	33.3 ± 5.0	34.5 ± 3.0
Plasma glucose (mmol/L)	5.7 ± 0.9	5.4 ± 0.5
NIHSS (score range 0–42)	0.1 ± 0.3	0.1 ± 0.3
mRS (score range 0–6)	0.9 ± 0.6	0.7 ± 0.6
MoCA (score range 0–30)	27.9 ± 1.9	28.8 ± 1.3

Results are shown as mean ± SD or an amount (n). BMI, body mass index; HbA1c, hemoglobin A1c; HDL, high-density lipoprotein; LDL, low-density lipoprotein; MoCA, Montreal Cognitive Assessment; mRS, modified Rankin Scale; NIHSS, National Institutes of Health Stroke Scale.

**TABLE 2:** Data of repeated measurements.

Outcome/Time	Baseline	30 min	60 min	90 min	120 min	150 min	180 min	*P*-value
TCD (cm/s) – exenatide	68.5 ± 11.4	70.9 ± 11.8	70.9 ± 12.5	68.2 ± 10.9	69.2 ± 12.7	68.8 ± 13.0	68.7 ± 12.1	0.058
TCD (cm/s) – placebo	61.8 ± 9.0	62.9 ± 12.2	62.2 ± 11.9	64.7 ± 14.3	65.3 ± 12.5	65.4 ± 11.4	65.8 ± 12.9	
sBP (mmHg) – exenatide	133.9 ± 19.4	–	134.8 ± 16.0	–	139.6 ± 16.2	–	137.5 ± 20.1	0.51
sBP (mmHg) – placebo	129.2 ± 10.2	–	139.0 ± 15.9	–	136.1 ± 12.2	–	135.4 ± 14.2	
dBP (mmHg) – exenatide	77.9 ± 11.3	–	82.1 ± 10.6	–	82.1 ± 11.7	–	80.3 ± 12.2	0.40
dBP (mmHg) – placebo	77.5 ± 6.1	–	81.8 ± 9.9	–	81.5 ± 8.8	–	80.7 ± 8.9	

**Outcome/Time**	**Baseline**	**15 min**	**45 min**	**75 min**	**105 min**	**135 min**	**165 min**	***P*-value**

Insulin (pmol/L) – exenatide	42.5 ± 47.3	65.2 ± 62.7	71.0 ± 57.1	48.0 ± 37.6	44.8 ± 44.8	42.1 ± 36.7	41.2 ± 31.0	0.0003
Insulin (pmol/L) – placebo	32.8 ± 19.6	37.9 ± 18.3	43.4 ± 24.6	35.2 ± 21.4	39.9 ± 24.9	35.0 ± 19.5	34.5 ± 18.0	
Plasma glucose (mmol/L) – exenatide	5.5 ± 0.5	5.4 ± 0.5	4.6 ± 0.7	4.4 ± 0.5	4.4 ± 0.5	4.7 ± 0.4	4.9 ± 0.3	0.0001
Plasma glucose (mmol/L) – placebo	5.4 ± 0.5	5.2 ± 0.4	5.3 ± 0.3	5.3 ± 0.5	5.2 ± 0.5	5.1 ± 0.4	5.2 ± 0.4	

Results are shown as mean ± SD. P-values are from the statistical repeated measurement analysis. dBP, diastolic blood pressure; sBP, systolic blood pressure; TCD, transcranial doppler.

**TABLE 3:** Data of secondary outcomes.

Outcome/Time	Baseline	180 min	*P*-value
ABI – exenatide	1.1 ± 0.1	1.1 ± 0.1	0.71
ABI – placebo	1.2 ± 0.1	1.2 ± 0.1	
**Plethysmography**			
RHI – exenatide	2.0 ± 0.5	2.2 ± 0.7	0.45
RHI – placebo	2.0 ± 0.3	2.3 ± 0.4	
AI@75 – exenatide	11.3 ± 14.1	12.8 ± 15.2	0.86
AI@75 – placebo	1.9 ± 12.6	4.5 ± 13.6	
**Biomarkers (pg/mL)**			
E-selectin – exenatide	4630 ± 1860	4050 ± 2500	0.31
E-selectin – placebo	4210 ± 2560	4350 ± 2920	
TNF – exenatide	1.84 ± 0.754	1.86 ± 0.707	0.33
TNF – placebo	1.56 ± 0.445	1.42 ± 0.412	
IL-6 – exenatide	0.792 ± 0.532	1.79 ± 0.928	0.11
IL-6 – placebo	0.762 ± 0.426	1.80 ± 1.144	
IL-1β – exenatide	0.067 ± 0.181	0.028 ± 0.095	0.34
IL-1β – placebo	0.008 ± 0.031	0.007 ± 0.027	
VCAM1 – exenatide	1,080,000 ± 317,000	1,090,000 ± 333,000	0.73
VCAM1 – placebo	1,170,000 ± 574,000	1,010,000 ± 372,000	
ICAM1 – exenatide	730,000 ± 143,000	748,000 ± 153,000	0.74
ICAM1 – placebo	694,000 ± 328,000	698,000 ± 306,000	

Results are shown as mean ± SD. P-values are from the statistical unpaired sampled t-test. ABI, ankle-brachial index; AI@75, augmentation index standardized to a heart rate of 75; ICAM1, intercellular adhesion molecule 1; IL, interleukin; RHI, regional hyperemia index; TCD, transcranial doppler; TNF, tumor necrosis factor; VCAM1, vascular cell adhesion molecule 1.

### Transcranial doppler

**FIGURE 2:**
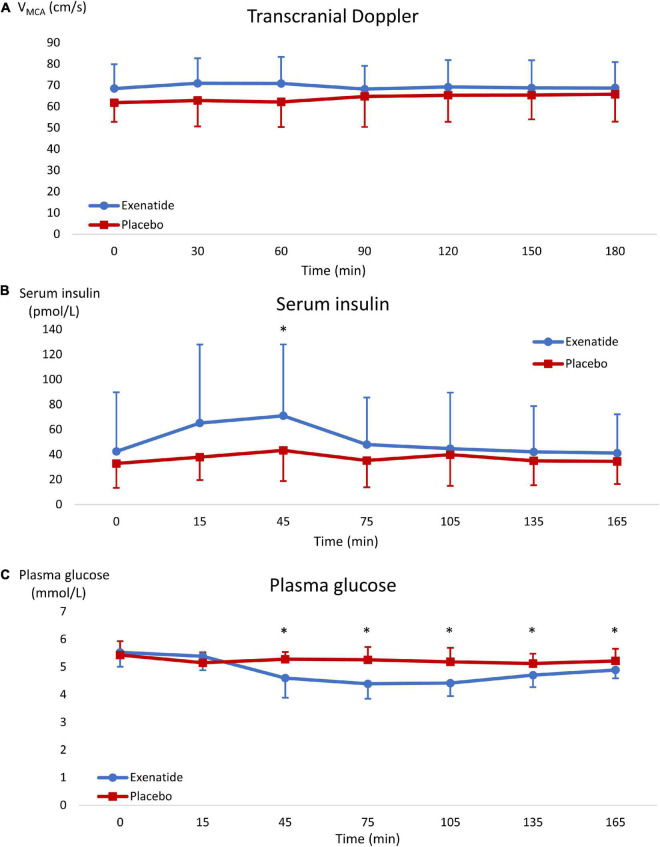
Results graphs. Results are shown as mean ± SD. Blue data series represent the exenatide group and red represents the placebo group. Graph **(A)** shows Transcranial Doppler, graph **(B)** shows serum insulin, and graph **(C)** shows plasma glucose. *, significant difference between groups.

Repeated measurements analyses of TCD showed no difference between treatment groups. Albeit non-significant, there was a trend toward a higher V_MCA_ in the active group compared to placebo during the first hour after administration (Figure 2 and Table 3).

### Plethysmography

There was no difference in peripheral arterial function between treatment groups comparing change from baseline and 180 min post-medication assessment (Table 3).

### Insulin, glucose, and biomarkers

There was a significant increase in measured insulin concentration at 45 min post-medication in the exenatide group compared to placebo with a mean difference of 28 **±** 61 pmol/L (*p* = 0.0003) (Figure 2). Correspondingly, there was a significant decrease in plasma glucose in the exenatide group, starting from 45 min post-medication throughout the whole trial day (*p* = 0.0001) with a mean difference of 0.61 **±** 0.41 mmol/L between groups (Figure 2). There was no difference in the measured biomarkers concentration from baseline to post-treatment between the groups (Table 3).

### Ankle-brachial index and blood pressure

There was no difference in ABI and blood pressure measurements between groups when comparing changes from baseline to 180 min post-medication (Table 3).

### Side effects

The main side effects reported after administration of exenatide were headache and gastro-intestinal discomfort, but no serious adverse advents were reported. All side effects were self-limited within one day. For further information on side effects, see Table 4.

**TABLE 4:** Side effects.

Side effects	Exenatide	Placebo	*P*-value
Headache	8	5	0.28
Dizziness	1	2	0.56
Increased temperature perception	1	0	0.33
Uneasy	1	0	0.33
Fatigue	3	2	0.64
Nausea	3	3	1
Abdominal pain	3	0	0.07
Flatulence	2	0	0.15

Table over registered side effects. Results are shown as the number of subjects who experienced the specific side effects in the different treatment groups. P-values are from the statistical unpaired sampled t-test.

## Discussion

GLP-1RAs show increasing potential as neuroprotective and stroke prophylactic agents due to positive results on stroke incidence in GLP-1RA treated patients with diabetes (Bellastella et al., 2020). However, little is known on potential mechanisms of action. In this randomized, double-blind, placebo-controlled parallel-arm pilot study, we found no effect of exenatide on cerebral and peripheral vasculature or on inflammatory biomarkers in healthy elderly volunteers. A repeated measurement analysis showed a near-significant difference between groups with an increase in V_MCA_ in the exenatide group. However, this most likely reflects a difference in baseline values between groups, where V_MCA_ was higher in the exenatide-group at baseline.

Our study is the first to assess effect of GLP-1RA on human cerebral and peripheral vasculature as well as inflammatory response in non-diabetic subjects. Since this is a pilot trial, some limitations may apply. First, it is a small pilot study with 15 subjects in each treatment arm. Though previously shown to detect small changes, TCD may be sensitive to head or hand movement. We tried to reduce risk of variations by optimizing the signal to the same anatomical area for each measurement. Second, we did not assess end-tidal carbon dioxide concomitant to TCD measurements. Changes in end-tidal carbon dioxide is known to affect V_MCA_ (Cencetti et al., 1997). However, all individuals in the trial were awake and relaxed in supine position during measurements, thus no change in breathing pattern and end-tidal carbon dioxide was expected.

When we applied TCD measurements, we assessed the CBF velocity in the large arteries in the brain. A change in velocity is an expression of a change in the vascular tone and can be used as a surrogate for blood flow, but only when assuming that the caliber of larger vessels is constant (Kontos, 1989). We found no effect of a single dose exenatide on the MCA indicating that GLP-1RA does not immediately change large artery diameter. However, a single dose of GLP-1RA may not reflect possible long-term vascular changes, which requires further evaluation. In this study, we did not assess possible effects on the cerebral microvasculature, which could be the target for GLP-1RAs in the cerebral vasculature (Chai et al., 2012). To identify general or regional brain microvascular changes in humans, other measurements could be applied, e.g., arterial spin labeling magnetic resonance imaging (MRI) or H_2_O positron emission tomography (PET). The lack of vascular effects of GLP-1RA in this trial, may relate to inclusion of healthy aging subjects. In both pre-clinical and clinical studies, GLP-1 seemed to improve endothelial dysfunction only with concomitant hyperglycemia, as reported in studies on rat mesenteric arteries (Salheen et al., 2015; Bangshaab et al., 2019) and subcutaneous human arterioles (Koska et al., 2015). We only included individuals without diabetes, and no individuals were observed to have hyperglycemia during the trial, as they were observed during fasting to reduce variance in outcome. This may result in a possible missed hyperglycemic-dependent vascular effect. Exenatide as single dose was found not to have immediate effect on blood pressure. While others also found no acute effect of GLP-1RA on blood pressure (Goud et al., 2016), long-term treatment GLP-1RA treatment had a minor blood pressure reducing effect (Wang et al., 2013; Sun et al., 2015). We found no effect of exenatide on peripheral arterial function measured with EndoPAT. We have previously shown a substantial consistency on day-to-day measurements with EndoPAT but less consistency on same-day measurements, which may be reflected in our findings of no change (Hansen et al., 2017; Ölmestig et al., 2020). ABI is used to estimate peripheral artery disease with analysis of the systolic blood pressure in the ankle divided by the systolic blood pressure in the arm. We found no effect of exenatide on ABI. Since GLP-1RAs had no acute effect on blood pressure, it is less likely to have acute effects on ABI measurements.

It has recently been suggested that GLP-1RA acts as an anti-inflammatory drug (Lee and Jun, 2016). We found that a single dose of exenatide did not change levels of endothelial and inflammatory biomarkers. The lack of effect on inflammatory parameters in this study is probably due to the inclusion of healthy individuals without vascular or chronic inflammatory disease. In contrast to this, previous studies using multiple doses of GLP-1RAs found reductions in circulating pro-inflammatory biomarkers in both animals and humans with diabetes (Lee and Jun, 2016; Zobel et al., 2021). Inflammation appears to play an important role in several acute and chronic diseases, including vascular diseases and diabetes and could be a potential treatment target (Lee and Jun, 2016). In animal models of acute stroke, GLP-1RA reduce TNF and IL-6 levels (Marlet et al., 2018), both pro-inflammatory cytokines involved in stroke pathology (Wilkins and Swerdlow, 2015; Lambertsen et al., 2019). Increased TNF levels have also been confirmed in one human stroke study (Bokhari et al., 2014).

The side-effects of exenatide are few, and it is safe to use in diabetic patients (Knop et al., 2017). We found exenatide to have few and non-severe side-effects in this population of healthy elderly subjects. Most reported side-effects were headache and gastro-intestinal discomfort; however, these were also seen to some degree in the placebo group and may represent an adverse event linked to the trial day and fasting and not the trial drug.

GLP-1RAs have shown to induce vasodilation and increase myocardial blood flow in patients with type 2 diabetes, however the mechanisms by which this occur are poorly understood (Gejl et al., 2012). Bangshaab et al. (2019) investigated a potential mechanism behind the vasodilatory properties of GLP-1RAs. They found that stimulation of the GLP-1 receptors by liraglutide was directly involved in relaxation of branched mesenteric arteries but not arteries without branches in rats (Bangshaab et al., 2019). They further found that GLP-1 improved endothelial function in arteries exposed to hyperglycemia but not normoglycemia (Bangshaab et al., 2019). These findings indicate that the target of GLP-1RAs may be branched arteries and that the vasodilatory effect is mainly seen during hyperglycemia, and possibly also why GLP-1RAs tend to reduce blood pressure in patients with diabetes (Wang et al., 2013; Sun et al., 2015; Goud et al., 2016). However, this effect is often seen after a longer treatment period (Wang et al., 2013; Sun et al., 2015; Goud et al., 2016), indicating that the pharmacological action of GLP-1RA on human vasculature might only present during continuous treatment. It would therefore be of interest to investigate if long-term treatment with a GLP-1RA can influence the cerebral vasculature. However, the effect of GLP-1RAs on vasculature is limited and probably does not fully explain the neuroprotective effect seen in animals and patients with diabetes. The neuroprotective mechanism of GLP-1RAs could be independent of vascular tone and involve other pharmacological actions of GLP-1, e.g., the anti-inflammatory properties (Lee and Jun, 2016) as previously mentioned.

## Conclusion and perspectives

This trial investigated the effect and feasibility of a single dose of exenatide (5 μg) on cerebral and peripheral arterial function in healthy elderly volunteers. Exenatide was safe and feasible to use, but it had no acute effect on mean CBF velocity in the MCAs or on peripheral arteries. Previous studies have shown that GLP-1RAs directly affect vasculature, however, we believe we may overlook potential vascular effects by the methods used. Additional studies on the effect of GLP-1RAs on both global and regional CBF are needed, as well as investigation of the cerebral microvasculature, to shed light on the neuroprotective mechanisms behind GLP-1RAs. Finally, it is necessary to investigate this medication in patients with cerebrovascular disease, since GLP-1RAs might act differently in patients with vascular pathology and endothelial dysfunction.

## Data availability statement

The raw data supporting the conclusions of this article will be made available by the authors, without undue reservation.

## Ethics statement

The studies involving human participants were reviewed and approved by the Ethics Committee in the Capital Region of Denmark (H-16022532). The patients/participants provided their written informed consent to participate in this study.

## Author contributions

IM, JÖ, and CK conceived the original data. IM, TV, JR, and CK designed the study. KL performed the analysis of biomarkers. ER performed the statistical analysis. JÖ wrote the manuscript. All authors contributed to the drafts and approved the final version.

## Footnotes

1
www.forsoegsperson.dk

## Abbreviations

ABI: ankle brachial index
AI@75: augmentation index standardized to a heart rate of 75
CBF: cerebral blood flow
CV: coefficient of variation
GCP: good clinical practice
GLP-1: glucagon-like peptide 1
GLP-1RA: glucagon-like peptide 1 receptor agonist
ICAM1: intracellular adhesion molecule 1
IL: interleukin
MoCA: Montreal cognitive assessment
MCA: middle cerebral artery
MRI: magnetic resonance imaging
mRS: modified Rankin scale
NIHSS: national institute of health stroke scale
PET: positron emission tomography
RHI: regional hyperemia index
TCD: transcranial doppler
TNF- α: tumor necrosis factor alpha
VCAM: vascular cell adhesion molecule
V_MCA_: mean blood flow velocity in the middle cerebral artery.
