# Supplementary material for: A single dose of exenatide had no effect on blood flow velocity in the middle cerebral artery in elderly healthy volunteers: Randomized, placebo-controlled, double-blind clinical trial
Source: Front Aging Neurosci. 2022 Jul 25;14:899389. doi: 10.3389/fnagi.2022.899389 (PMC9831269; doi:10.3389/fnagi.2022.899389)
Supplement: Supplementary file 1 [file Data_Sheet_1.DOCX]

**Supplementary material 1**

Inclusion criteria:

1. Age ≥ 50 years
2. Has given written informed consent

Exclusion criteria:

1. Intracerebral hemorrhage
2. Subdural / epidural hemorrhage
3. Subarachnoid hemorrhage
4. Previously major structural damage to the brain (e.g. sequelae after large stroke or brain surgery)
5. Type 1 diabetes
6. Type 2 diabetes
7. Known atrial fibrillation
8. Known stenosis of the carotid arteries above 50 %
9. Known allergy to GLP-1RA
10. Hepatic impairment (ALT > 3 x upper normal limit)
11. Renal impairment (eGFR < 30 ml/min)
12. Inflammatory bowel disease
13. Previous pancreatitis
14. Heart failure (NYHA class 3-4)
15. Pregnancy or lactation
16. Patient is not expected to co-operate according to trial specific investigations
17. Visualization of the middle cerebral artery bilaterally by transcranial Doppler is not possible
